# Endoscopic Ultrasound-Guided Hepaticogastrostomy via a Retrosternal Gastric Tube Conduit for Obstructive Cholangitis Caused by Ampullary Carcinoma: A Case Report

**DOI:** 10.7759/cureus.112016

**Published:** 2026-07-03

**Authors:** Koji Takahashi, Masataka Nakano, Taiga Sudo, Hidehiro Kamezaki

**Affiliations:** 1 Gastroenterology, Chiba University, Chiba, JPN; 2 Pathology, Eastern Chiba Medical Center, Togane, JPN; 3 Gastroenterology, Eastern Chiba Medical Center, Togane, JPN

**Keywords:** acute cholangitis, eus-hgs, plastic stent, retrosternal gastric tube reconstruction, subtotal esophagectomy

## Abstract

Subtotal esophagectomy with retrosternal gastric tube reconstruction alters upper gastrointestinal anatomy and can create two obstacles to endoscopic retrograde cholangiopancreatography (ERCP): displacement and rotation of the pylorus and proximal duodenum, which impede side-viewing duodenoscopy, and ampullary carcinoma, which may obscure the bile duct orifice and prevent selective cannulation. When endoscopic drainage fails, endoscopic ultrasound-guided hepaticogastrostomy (EUS-HGS) offers an alternative route for internal biliary decompression. Reports of EUS-HGS performed from within a retrosternal gastric tube conduit are limited. A 79-year-old woman with prior subtotal esophagectomy and retrosternal gastric tube reconstruction presented with acute cholangitis and obstructive jaundice caused by ampullary adenocarcinoma. ERCP was abandoned because tumor infiltration obscured the bile duct orifice. EUS-HGS was performed on the same day through the gastric conduit. A 19-gauge puncture and a double-guidewire technique enabled placement of a 7-Fr, 14-cm plastic stent without adverse events. Cholangitis resolved, diet was resumed four days later, and the stent remained patent at 57 days. Histology confirmed adenocarcinoma. EUS-HGS via a retrosternal gastric tube conduit was feasible and achieved internal biliary drainage in a patient with complex post-esophagectomy anatomy and failed ERCP.

## Introduction

Endoscopic retrograde cholangiopancreatography (ERCP) is the standard method for biliary decompression in obstructive jaundice, but it requires both endoscopic access to the papilla and successful cannulation of the bile duct; when either is not achievable, an alternative approach is needed [1,2].

Subtotal esophagectomy with gastric tube reconstruction, the standard operation for resectable esophageal cancer, fashions the stomach into a narrow tubular conduit transposed to the neck via the retrosternal or posterior mediastinal route [3]. The resulting displacement and rotation of the pylorus and proximal duodenum make side-viewing duodenoscopy difficult [4]. Ampullary carcinoma adds a further obstacle: when an invasive tumor replaces the normal ampullary architecture, the bile duct orifice may be obscured, and selective cannulation may be impossible even when the papilla is reached [5]. Percutaneous transhepatic biliary drainage (PTBD) has traditionally served as rescue therapy in this situation, but its external drain and associated morbidity are considerable in frail oncology patients [6].

Endoscopic ultrasound-guided hepaticogastrostomy (EUS-HGS) is a less invasive internal alternative to PTBD, with high technical success reported in prospective and multicenter studies [7-11]. Reports of EUS-HGS performed from within a retrosternal gastric tube conduit-a configuration in which the entire stomach forms the conduit and no bowel interposition is present-remain limited, and this anatomy poses specific challenges: reduced intraluminal working space, an altered conduit-to-liver relationship, and a potential risk of mediastinal injury during transmural puncture. Here, we describe a case in which EUS-HGS was successfully performed through a retrosternal gastric tube conduit after failed ERCP.

## Case presentation

A 79-year-old woman was referred for urgent biliary drainage. Her history included subtotal esophagectomy with retrosternal gastric tube reconstruction (six years earlier) and sigmoid colon cancer resection (six years earlier). She had severe frailty (Eastern Cooperative Oncology Group {ECOG} Performance Status 3) [12].

Three days before the procedure, jaundice was identified at a routine outpatient visit to the referring hospital, where she was admitted the same day. Computed tomography (CT) showed an ampullary tumor with upstream bile duct dilatation. Fever developed one day before the procedure, and she was transferred to our institution on the day of the procedure for endoscopic drainage. On arrival, she was febrile with chills and rigors, meeting the criteria for acute cholangitis according to the Tokyo Guidelines 2018 [13], and her septic presentation required prompt biliary decompression. Pre-procedure laboratory values are summarized in Table 1.

**Table 1:** Pre-procedure laboratory values. Alb, albumin; ALP, alkaline phosphatase; ALT, alanine aminotransferase; APTT, activated partial thromboplastin time; AST, aspartate aminotransferase; BUN, blood urea nitrogen; CA19-9, carbohydrate antigen 19-9; CEA, carcinoembryonic antigen; Cl, chloride; Cre, creatinine; CRP, C-reactive protein; D-Bil, direct bilirubin; DD, D-dimer; GGT, gamma-glutamyl transferase; Glu, glucose; Hb, hemoglobin; HbA1c, glycated hemoglobin; Ht, hematocrit; K, potassium; LD, lactate dehydrogenase; MCH, mean corpuscular hemoglobin; MCHC, mean corpuscular hemoglobin concentration; MCV, mean corpuscular volume; Na, sodium; NT-proBNP, N-terminal pro-B-type natriuretic peptide; Plt, platelet count; PT, prothrombin time; PT-INR, prothrombin time-international normalized ratio; RBC, red blood cell count; T-Bil, total bilirubin; TP, total protein; WBC, white blood cell count. H, above upper reference limit; L, below lower reference limit.

Parameter	Value	Reference range	Unit	Flag
WBC	11,510	3,300-8,600	/μL	H
RBC	284	386-492	×10⁴/μL	L
Hb	8.7	11.6-14.8	g/dL	L
Ht	25.0	35.1-44.4	%	L
Plt	49.7	15.8-34.8	×10⁴/μL	H
MCV	88.0	84.0-98.0	fL	-
MCH	30.6	28.0-33.0	pg	-
MCHC	34.8	32.0-35.0	%	-
PT	86	68-114	%	-
PT-INR	1.08	0.90-1.16	-	-
APTT	26.8	26.5-38.1	s	-
DD	1.6	≤1.0	μg/mL	H
T-Bil	6.1	0.4-1.5	mg/dL	H
D-Bil	4.4	0.0-0.2	mg/dL	H
AST	143	13-30	U/L	H
ALT	117	7-23	U/L	H
ALP	829	43-115	U/L	H
GGT	861	9-32	U/L	H
LD	181	125-220	U/L	-
TP	5.5	6.6-8.1	g/dL	L
Alb	2.2	4.1-5.1	g/dL	L
Glu	138	73-109	mg/dL	H
HbA1c	6.0	4.9-6.0	%	-
BUN	10.4	8.0-20.0	mg/dL	-
Cre	0.43	0.46-0.79	mg/dL	L
Na	132	138-145	mmol/L	L
K	4.3	3.6-4.8	mmol/L	-
Cl	98	101-108	mmol/L	L
CRP	8.60	≤0.14	mg/dL	H
CA19-9	178.6	≤36.8	U/mL	H
CEA	2.0	≤5.2	ng/mL	-
NT-proBNP	781.6	≤54.5	pg/mL	H

Contrast-enhanced CT demonstrated marked intrahepatic bile duct dilation with an ampullary mass (Figure 1), and the gastric tube was identified in the retrosternal position.

**Figure 1:**
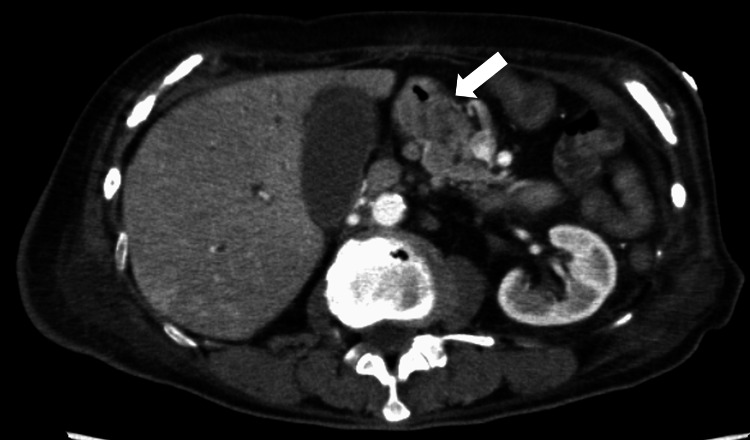
Contrast-enhanced computed tomography image showing the ampullary tumor and biliary obstruction. An ill-defined soft-tissue mass (arrow) approximately 28 mm in diameter is present at the level of the duodenal papilla, with marked upstream intrahepatic and extrahepatic bile duct dilatation, consistent with malignant ampullary obstruction.

Same-day biliary drainage was indicated. Conscious sedation was achieved with intravenous pentazocine, hydroxyzine pamoate, and midazolam. Initial endoscopy with a forward-viewing endoscope showed a tumor at the duodenal papilla, and biopsy specimens were obtained. An oblique-viewing duodenoscope was advanced through the gastric conduit to the duodenum, and the ampullary region was visualized. The tumor had replaced the normal ampullary architecture, and the bile duct orifice could not be identified (Figure 2). Selective cannulation was not possible, and ERCP was abandoned.

**Figure 2:**
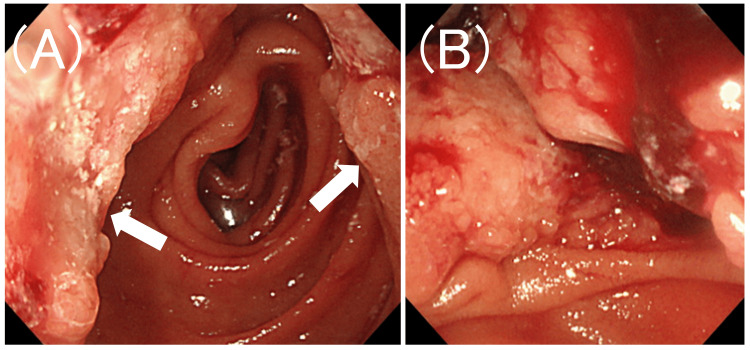
Endoscopic appearance of the ampullary tumor. (A) Forward-view endoscopic image obtained from within the retrosternal gastric conduit during the initial examination. An irregular, hemorrhagic, elevated, friable tumor (arrow) occupies the area of the ampulla of Vater, consistent with invasive carcinoma. Forceps biopsy was performed before attempting endoscopic retrograde cholangiopancreatography (ERCP). (B) Oblique-view duodenoscopic image of the same lesion. The tumor has replaced the normal papillary architecture; neither the bile duct orifice nor any residual papillary prominence is identifiable, which precluded selective deep cannulation and required abandonment of ERCP.

EUS-HGS was then performed in the same session. The patient was placed in the prone position, and CO2 insufflation was used throughout to limit the consequences of any gas leak into the mediastinum. A curved linear echoendoscope (GF-UCT260; Olympus, Tokyo, Japan) was advanced through the gastric conduit. At a level just below the diaphragm-where the conduit lies closest to the hepatic parenchyma before ascending into the thoracic inlet-the dilated left lateral segment intrahepatic bile duct (B2/B3 junction) was identified by endosonography (Figure 3). Color Doppler confirmed the absence of intervening vessels along the intended puncture trajectory. The bile duct was punctured with a 19-gauge needle (EZShot3 Plus; Olympus, Tokyo, Japan), and bile aspiration confirmed correct positioning. A guidewire (CAPELLA; Medico's Hirata, Osaka, Japan) was advanced into the extrahepatic bile duct. An uneven double-lumen cannula (PIOLAX, Kanagawa, Japan) - a dual-channel catheter that allows simultaneous cholangiography and placement of a second guidewire - was inserted for cholangiography, followed by placement of a second guidewire (VisiGlide2; Olympus, Tokyo, Japan) to establish a double-guidewire technique. This technique stabilizes the scope position and reduces the risk of guidewire dislodgement during tract dilation and stent deployment. The tract was dilated with a 7-Fr ES dilator (Zeon Medical, Tokyo, Japan) alone; neither balloon nor electrocautery dilation was required. A 7-Fr, 14-cm plastic stent (Through&Pass TYPE IT; Gadelius Medical, Tokyo, Japan) was then deployed across the hepaticogastric tract, with bile observed draining into the conduit lumen.

**Figure 3:**
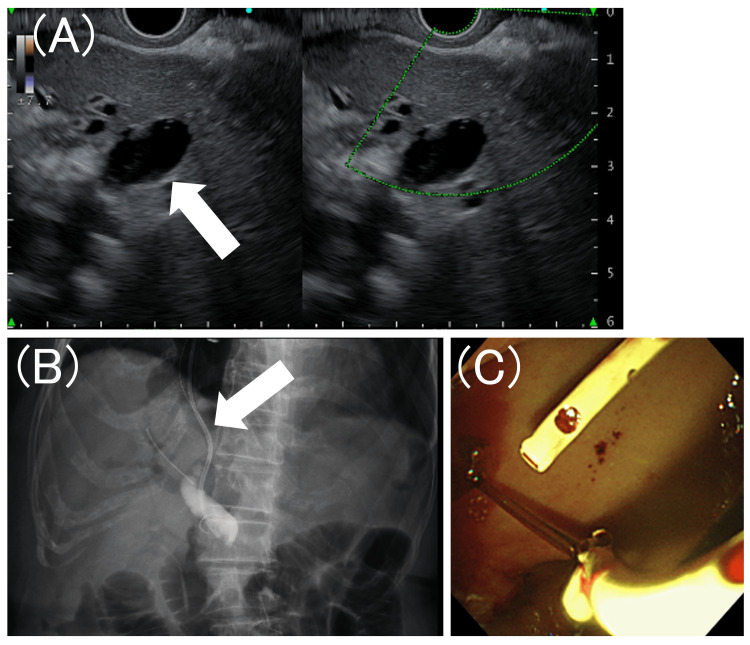
Procedural images of endoscopic ultrasonography-guided hepaticogastrostomy. (A) Endosonographic views from within the retrosternal gastric conduit at the infradiaphragmatic level. Left panel (B-mode): the dilated left lateral segment intrahepatic bile duct at the B2/B3 junction (arrow) is identified adjacent to the conduit wall, providing a suitable puncture target. Right panel (color Doppler overlay): the absence of blood flow in the planned puncture corridor between the conduit wall and the bile duct confirms a vessel-free trajectory. (B) Fluoroscopic image during endoscopic ultrasound-guided hepaticogastrostomy. After 19-gauge needle puncture and bile aspiration, a guidewire was coiled within the biliary system and a second guidewire was placed through the uneven double-lumen cannula; the tract was then dilated and a 7-Fr, 14-cm through-and-pass plastic stent (arrow) was deployed across the hepaticogastric fistula. (C) Endoscopic confirmation of stent placement, with bile draining into the gastric lumen from the gastric flap of the stent.

CT performed the day after the procedure confirmed satisfactory stent positioning (Figures 4, 5). No procedure-related complications occurred. Intravenous antibiotics were continued until defervescence, and oral intake was withheld until day 4, when clinical and biochemical improvement was confirmed. Fever resolved within two days, and jaundice improved progressively.

**Figure 4:**
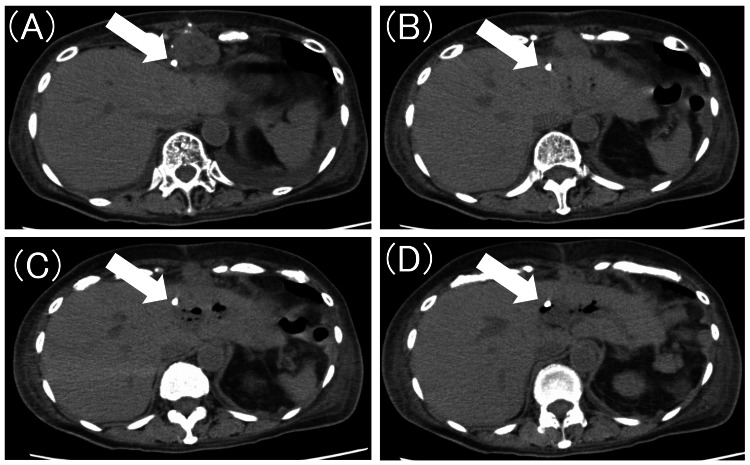
Contrast-enhanced computed tomography images, axial views (day 1 after the procedure), confirming stent position along its intrahepatic course. (A) The stent entry point (arrow) is at the infradiaphragmatic conduit wall, within the abdominal cavity, with no pneumomediastinum or pneumothorax. (B) The stent penetrates the hepatic capsule (arrow) and enters the liver parenchyma at the target site. (C) The intrahepatic stent course (arrow) traverses the periductal liver parenchyma of the left lateral segment without perihepatic fluid collection or hematoma. (D) The stent tip (arrow) lies within the dilated intrahepatic bile duct; the duct lumen is almost entirely replaced by air, consistent with effective decompression.

**Figure 5:**
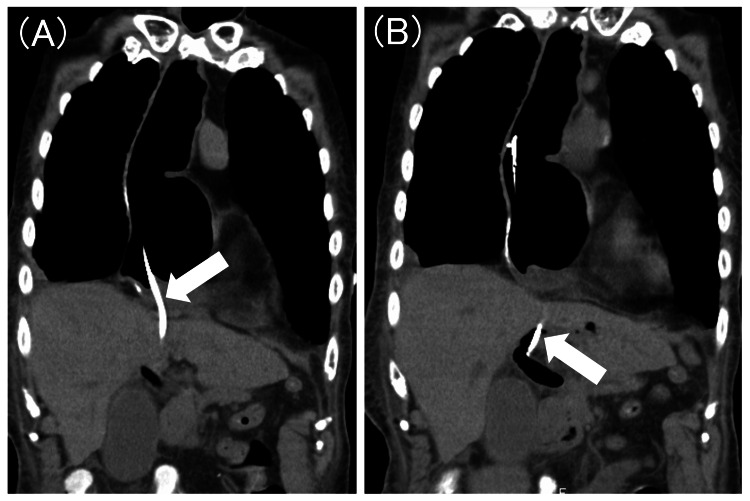
Contrast-enhanced computed tomography images, coronal views (day 1 after the procedure), showing the overall stent trajectory from the gastric conduit to the intrahepatic biliary tree. (A) The stent is inserted from the entry site (arrow) in the gastric tube wall toward the intrahepatic bile duct. (B) The stent tip (arrow) lies within the bile duct.

Histopathological analysis of the ampullary biopsies confirmed well-differentiated adenocarcinoma (Figure 6). The patient's general condition had declined, and she spent most of her time in bed (ECOG Performance Status 3). After discussion with the medical team, she expressed her wish to forgo surgery and systemic chemotherapy and to receive palliative care. She was transferred back to the referring hospital nineteen days after the procedure. At transfer, jaundice had resolved, and serum bilirubin and hepatobiliary enzyme levels had normalized, indicating sustained biliary decompression. The patient was subsequently transferred to a further facility 57 days after stent placement; at that time, the stent remained patent, with no clinical or biochemical evidence of recurrent cholangitis or stent occlusion.

**Figure 6:**
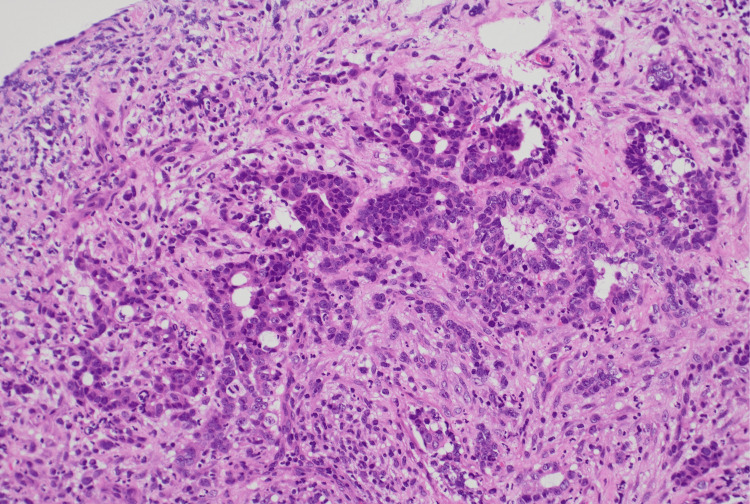
Histopathological findings of the ampullary biopsy specimen (hematoxylin and eosin stain, ×200). Atypical columnar epithelial cells proliferate in an irregular glandular pattern, forming tubules of varying caliber. The neoplastic cells have enlarged, round-to-oval nuclei with coarse chromatin and identifiable nucleoli, consistent with well-differentiated adenocarcinoma.

## Discussion

Reports of EUS-HGS performed from within a retrosternal gastric tube conduit after subtotal esophagectomy are limited, and this case adds procedural and anatomical detail to that small body of evidence. In the configuration described here, the stomach forms a narrow tubular conduit in the anterior mediastinum, and the echoendoscope operates from within it. This differs from previously reported EUS-guided biliary drainage scenarios [14,15]. Understanding why this anatomy is difficult, and how each technical choice addressed those difficulties, is useful if the approach is to be extended to other patients.

In conventional EUS-HGS, the procedure is performed from a normally positioned stomach with adequate intraluminal working space and a well-characterized relationship to the left hepatic lobe [14,15]. The retrosternal gastric tube, by contrast, lacks normal mesenteric attachments, is oriented vertically within the anterior mediastinum, is narrower, and faces the liver anteriorly rather than posteriorly-features that together limit scope maneuverability and the range of available puncture angles. In this case, an adequate acoustic window was identified only at the infradiaphragmatic level, where the conduit passes closest to the hepatic parenchyma before ascending into the thoracic inlet. Identifying this window was the primary objective of the initial endosonographic survey; whether the infradiaphragmatic window is consistently available will depend on reconstruction type and body habitus and cannot be generalized from a single case. Pre-procedural annotation of contrast-enhanced CT to define the diaphragmatic plane and the conduit-to-liver relationship was helpful in this case and seems a reasonable planning step, as it confirms anatomical feasibility and allows the operator to rehearse the puncture trajectory before scope insertion.

Three precautions were taken to reduce the risk of complications. First, pre-procedural CT confirmed that the hepatic parenchyma was in direct contact with the infradiaphragmatic conduit wall at the planned puncture site, so that the needle trajectory remained within the abdominal cavity. Second, color Doppler interrogation of the target segment immediately before puncture excluded interposing vessels. Third, the puncture was restricted to the abdominal segment of the conduit. The need for case-by-case anatomical assessment before EUS-guided biliary drainage in complex post-surgical anatomy has been described in comparative and indication studies [16,17].

A further consideration specific to this configuration is the direction of bile duct access. When the peripheral bile duct in the lateral segment is targeted, the echoendoscope points to the left, and puncture of a peripheral branch from this angle produces a steep angle between the puncture direction and the duct oriented toward the proximal side, which makes it difficult, and sometimes impossible, to advance the guidewire toward the hepatic hilum, and consequently makes stent placement difficult. To avoid this, a more central segment (the B2/B3 junction) was targeted, which allowed the guidewire to be advanced antegradely toward the common bile duct. The double-guidewire technique, made possible by the uneven double-lumen cannula for simultaneous cholangiography and second-wire placement, helped maintain scope stability and reduced the risk of wire dislodgement during tract dilation and stent deployment. Given the narrow conduit lumen and the constrained scope position, even minor scope displacement would have been difficult to correct, and the double-guidewire approach provided an added margin of safety.

The choice of stent in this setting requires consideration. In conventional EUS-HGS, a fully covered self-expandable metal stent (FC-SEMS) is often preferred because of its longer patency [18], and a dedicated plastic stent for EUS-HGS has also been reported as feasible and effective [19]. In this case, two anatomical factors favored a plastic stent. First, puncture at a relatively central ductal segment was required; deploying an FC-SEMS from this position could occlude the orifices of segmental branch ducts peripheral to the puncture site and lead to segmental cholangitis or hepatic parenchymal injury, a complication less likely in conventional EUS-HGS, where puncture is usually made more peripherally. Second, the narrow vertical orientation of the retrosternal conduit limits reliable caudal deflection of the gastric flange of an FC-SEMS; if the flange remained oriented orally rather than curling caudally, food could enter the biliary tract and predispose to recurrent stent-related cholangitis. The 7-Fr through-and-pass plastic stent, with its smaller caliber and simple linear geometry, avoids both concerns. The main trade-offs-shorter patency and the possible need for elective stent exchange-were judged acceptable given the patient's advanced performance status, her preference for palliative management, and her limited life expectancy. In patients requiring longer patency, a fully covered metal stent may still be preferable, and the choice should remain case-by-case. In the present case, the plastic stent remained patent for at least 57 days.

The novelty of the present case lies in the access route: unlike previously reported EUS-guided biliary drainage in post-esophagectomy anatomy, here the entire stomach formed a narrow conduit within the anterior mediastinum and the echoendoscope operated from within that conduit, with no residual native stomach and no interposed bowel available as a working station. Yane et al. described EUS-HGS after subtotal esophagectomy, but the reconstruction used free jejunal interposition, and the echoendoscope was operated from a residual stomach accessed with a colonoscope navigating the jejunal limb [20]. Hedjoudje et al. reported transcolonic EUS-guided biliary drainage after esophagogastrectomy with colon interposition, an anatomically distinct configuration in which the colon, not the gastric tube, served as the luminal access route [21]. In neither prior case was the entire stomach the conduit, and in neither was the echoendoscope required to operate within a narrow retrosternal tube. Together, these three cases illustrate the adaptability of EUS-guided biliary drainage to varied surgically reconstructed anatomy, and they indicate that each configuration requires individualized anatomical analysis rather than a uniform technique.

Several clinical features of this case merit comment. The patient's general weakness (ECOG Performance Status 3), multiple comorbidities (post-esophagectomy, post-colectomy), and abnormal liver function (total bilirubin 6.1 mg/dL, AST 143 U/L, ALP 829 U/L, albumin 2.2 g/dL) indicated that prompt biliary decompression was desirable. EUS-HGS was completed in the same endoscopic session immediately after ERCP failure, which avoided transfer to interventional radiology for PTBD and the associated pain, external drain, and burden in a patient who ultimately chose palliative care. Resolution of fever within two days and normalization of bilirubin and hepatobiliary enzymes by transfer indicate that the clinical objective was achieved.

This report has the limitations inherent to a single case. No general conclusions about success rate, safety, or optimal stent choice for EUS-HGS via a retrosternal gastric conduit can be drawn from one case. Technical success here may partly reflect operator experience and favorable individual anatomy rather than reproducible procedural characteristics. Stent patency was documented for approximately two months, but longer-term patency and the eventual need for stent exchange are unknown. Further cases are needed to determine whether the infradiaphragmatic acoustic window is consistently identifiable, whether plastic stents provide adequate long-term palliation in this setting, and whether the mediastinal complication risk remains low. Until such data are available, this approach is best reserved for centers with expertise in EUS-guided biliary drainage, and each case is best preceded by CT-based anatomical planning.

## Conclusions

In this patient with failed ERCP, EUS-HGS performed through a retrosternal gastric tube conduit after subtotal esophagectomy was feasible and achieved internal biliary drainage that remained effective at 57 days. The infradiaphragmatic segment of the conduit provided a reliable acoustic window for puncture in this case. Cholangitis resolved, oral intake was resumed, and sustained biliary decompression was confirmed at transfer, allowing the patient to pursue her preferred palliative course without an external drain.

Several technical considerations underpinned this outcome: pre-procedural CT-based anatomical planning to confirm conduit-to-liver contact and rehearse the puncture trajectory, restriction of the puncture to the abdominal segment of the conduit to avoid mediastinal injury, the double-guidewire technique to stabilize scope and guidewire position within the narrow lumen, and selection of a plastic stent when conduit geometry precludes a fully covered metal stent. Although no general conclusions can be drawn from a single case, this experience suggests that EUS-guided biliary drainage may be applicable to a broader range of complex post-surgical anatomies and supports its consideration at experienced centers when conventional drainage fails.

## References

[1] Dumonceau JM, Tringali A, Papanikolaou IS (2018). Endoscopic biliary stenting: indications, choice of stents, and results: European Society of Gastrointestinal Endoscopy (ESGE) Clinical Guideline - Updated October 2017. Endoscopy.

[2] Artifon EL, Aparicio D, Paione JB (2012). Biliary drainage in patients with unresectable, malignant obstruction where ERCP fails: endoscopic ultrasonography-guided choledochoduodenostomy versus percutaneous drainage. J Clin Gastroenterol.

[3] Watanabe M, Takahashi N, Tamura M (2024). Gastric conduit reconstruction after esophagectomy. Dis Esophagus.

[4] Sundaram S, Kale A (2023). Endoscopic ultrasound guided biliary drainage in surgically altered anatomy: a comprehensive review of various approaches. World J Gastrointest Endosc.

[5] Chathadi KV, Khashab MA, Acosta RD (2015). The role of endoscopy in ampullary and duodenal adenomas. Gastrointest Endosc.

[6] Winick AB, Waybill PN, Venbrux AC (2001). Complications of percutaneous transhepatic biliary interventions. Tech Vasc Interv Radiol.

[7] Bories E, Pesenti C, Caillol F, Lopes C, Giovannini M (2007). Transgastric endoscopic ultrasonography-guided biliary drainage: results of a pilot study. Endoscopy.

[8] Marx M, Caillol F, Autret A (2022). EUS-guided hepaticogastrostomy in patients with obstructive jaundice after failed or impossible endoscopic retrograde drainage: a multicenter, randomized phase II Study. Endosc Ultrasound.

[9] Paik WH, Lee TH, Park DH (2018). EUS-guided biliary drainage versus ERCP for the primary palliation of malignant biliary obstruction: a multicenter randomized clinical trial. Am J Gastroenterol.

[10] Park DH, Koo JE, Oh J (2009). EUS-guided biliary drainage with one-step placement of a fully covered metal stent for malignant biliary obstruction: a prospective feasibility study. Am J Gastroenterol.

[11] Kawakubo K, Isayama H, Kato H (2014). Multicenter retrospective study of endoscopic ultrasound-guided biliary drainage for malignant biliary obstruction in Japan. J Hepatobiliary Pancreat Sci.

[12] Oken MM, Creech RH, Tormey DC, Horton J, Davis TE, McFadden ET, Carbone PP (1982). Toxicity and response criteria of the Eastern Cooperative Oncology Group. Am J Clin Oncol.

[13] Kiriyama S, Kozaka K, Takada T (2018). Tokyo Guidelines 2018: diagnostic criteria and severity grading of acute cholangitis (with videos). J Hepatobiliary Pancreat Sci.

[14] Giovannini M, Moutardier V, Pesenti C, Bories E, Lelong B, Delpero JR (2001). Endoscopic ultrasound-guided bilioduodenal anastomosis: a new technique for biliary drainage. Endoscopy.

[15] Ogura T, Higuchi K (2016). Technical tips for endoscopic ultrasound-guided hepaticogastrostomy. World J Gastroenterol.

[16] Artifon EL, Marson FP, Gaidhane M, Kahaleh M, Otoch JP (2015). Hepaticogastrostomy or choledochoduodenostomy for distal malignant biliary obstruction after failed ERCP: is there any difference?. Gastrointest Endosc.

[17] Nakai Y, Isayama H, Yamamoto N (2017). Indications for endoscopic ultrasonography (EUS)-guided biliary intervention: does EUS always come after failed endoscopic retrograde cholangiopancreatography?. Dig Endosc.

[18] Imai H, Takenaka M, Omoto S (2017). Utility of endoscopic ultrasound-guided hepaticogastrostomy with antegrade stenting for malignant biliary obstruction after failed endoscopic retrograde cholangiopancreatography. Oncology.

[19] Umeda J, Itoi T, Tsuchiya T (2015). A newly designed plastic stent for EUS-guided hepaticogastrostomy: a prospective preliminary feasibility study (with videos). Gastrointest Endosc.

[20] Yane K, Seto K, Yoshida K, Hirokawa S, Morita K, Ikeda Y, Sumiyoshi T (2025). Endoscopic ultrasound-guided hepaticogastrostomy in a patient with complex postsurgical anatomy after subtotal esophagectomy. Endoscopy.

[21] Hedjoudje A, Jaïs B, Aubert A, Cattan P, Prat F (2021). EUS-guided drainage using hepaticocolostomy after esogastrectomy. VideoGIE.

